# Clinical and genetic features of *CNGA3* achromatopsia in preschool children: novel insights into retinal architecture and therapeutic window for clinical trials

**DOI:** 10.3389/fmed.2025.1560556

**Published:** 2025-04-02

**Authors:** Yanting Lai, Aohan Hou, Linyan Zhang, Limei Sun, Miner Yuan, Xiaoyan Ding

**Affiliations:** ^1^State Key Laboratory of Ophthalmology, Zhongshan Ophthalmic Center, Sun Yat-sen University, Guangzhou, China; ^2^Guangdong Provincial Key Laboratory of Ophthalmology and Visual Science, Guangdong Provincial Clinical Research Center for Ocular Diseases, Guangzhou, China

**Keywords:** achromatopsia, optical coherence tomography, novel variant, CNGA3, genotype-phenotype

## Abstract

**Purpose:**

Achromatopsia (ACHM) is a rare genetic disorder with an infantile onset that affects cone photoreceptors. This study aims to provide a comprehensive phenotyping of the retinal structure and identify novel genetic variants in a preschool cohort with ACHM in China.

**Methods:**

We recruited patients with pathogenic genes (*CNGA3, CNGB3, GNAT2, PDE6C, PDE6H*, and *ATF6*) known to cause ACHM, all of whom had an age of symptom onset before 6 years of age. Whole exome sequencing, Sanger sequencing, and comprehensive ocular examinations, including optical coherence tomography (OCT), were conducted. Furthermore, retinal outer layer damage was evaluated using a novel modified classification system.

**Results:**

Nystagmus (46.13%) and photophobia (46.13%) were the most common initial complaints/reports from parents of our patients. These symptoms are easily noticed early (mean age 0.88 ± 1.07 years at onset of initial symptom). OCT revealed a wide range of degeneration in the outer retina of the fovea, exactly in the interdigitation zone (IZ) and ellipsoid zone (EZ). Retinal outer layer damage was observed in 18 eyes (9 patients), with the modified classification distribution: grade 1 in 1 eye (5.6%), grade 2 in 9 eyes (50.0%), and grade 3 in 8 eyes (44.4%). Eleven novel variants of CNAG3 were identified. The higher grade of outer retinal layer damage was shown in patients with genetic variants, potentially leading to structural changes in the cyclic guanosine monophosphate (cGMP) binding site of the synthesized protein (*p* = 0.046).

**Conclusion:**

ACHM can manifest at very early stages of life. Mild damage to the outer layers of the retina is a typical change in early-stage ACHM. Patients with genetic variants potentially leading to structural changes in the cGMP binding site of the synthesized protein tend to exhibit more severe retinal phenotypes. Ultimately, our research may aid in formulating guidelines for selecting patients and determining the optimal timing for interventions in upcoming gene replacement therapies.

## 1 Introduction

Gene-therapeutic interventions hold significant promise for revolutionizing medicine, with ophthalmologic diseases affecting retinal function leading the way in this transformative journey. This groundbreaking potential has already been realized in the case of *RPE65*-related Leber congenital amaurosis, culminating in the recent approval of voretigene neparvovec (Luxturna, Spark Therapeutics, Inc., Philadelphia, USA), marking a milestone as the first gene therapy drug for an inherited retinal disease (1, 2). Another condition currently under the spotlight for gene supplementation approaches is autosomal recessive achromatopsia (ACHM), with both animal studies and several human phase I/II trials underway for ACHM related to *CNGA3* and *CNGB3* variants (NCT03758404 and NCT03001310, Farahbakhsh et al., NCT02935517, NCT02599922, NCT0261058212) (3–12).

ACHM, a rare genetic disorder with autosomal recessive inheritance, manifests as congenital dysfunction of the retinal cone photoreceptors, resulting in reduced visual acuity, nystagmus, photophobia, and absent or severely impaired color vision (13–15). The disease is primarily caused by gene variants encoding functional components of the phototransduction cascade. When light shines, the level of cyclic guanosine monophosphate (cGMP) decreases, causing cyclic nucleotide-gated (CNG) channels to close, the receptor to hyperpolarize, and ultimately inhibiting the release of glutamate. Specifically, variants in *CNGA3* and *CNGB3*, encoding the *α* and *β* subunits of the CNG channel in cone photoreceptors, collectively account for over 90% of cases. The remaining minority of cases are attributed to defects in genes regulating cGMP metabolism: *GNAT2* encodes cone transducin, a G-protein that activates phosphodiesterase 6 (PDE6); *PDE6C* and *PDE6H* constitute catalytic and inhibitory subunits of PDE6, respectively, which hydrolyze cGMP; while *ATF6*, operating at the endoplasmic reticulum, mediates stress-response pathways critical for photoreceptor survival (16–18).

Diagnosing ACHM hinges on the clinical presentation of symptoms, often evident from early infancy, including pendular nystagmus, photophobia, and diminishment or absence of color vision. While multimodal retinal imaging and electrophysiological assessments constitute standard diagnostic procedures, their implementation frequently encounters practical limitations in young children due to compliance challenges. Therefore, genetic testing supplements clinical diagnosis, aiding in identifying underlying genetic variants (19). Current management is limited to photophobia alleviation with tinted eyewear, low-vision aids, and genetic counseling (20). However, there is no curative treatment for ACHM, and gene therapy has proposed a new solution. Notably, the genetic landscape of ACHM exhibits significant regional and ethnic diversity, with variants in *CNGA3* predominant in certain populations, such as East Asian populations. Despite traditionally being viewed as a stationary cone dysfunction, emerging evidence suggests a progressive nature for ACHM. Considering the non-regenerative nature of retinal tissue, understanding the natural history of the disease, especially in the very early stage of life, is crucial to identifying optimal windows for intervention. In addition, early-stage assessment of photoreceptor damage severity and extent is pivotal in predicting the efficacy of gene therapy.

Thus, in this study, we present comprehensive phenotyping of retinal structure and novel genetic variants within a toddler-aged ACHM pediatric cohort in China. Our findings, in conjunction with existing research, lay a robust foundation for elucidating essential elements of retinal structure and functionality in ACHM. This is vital for refining candidate selection criteria and timing of interventions in anticipation of forthcoming gene replacement therapies targeting this debilitating disorder.

## 2 Methods

### 2.1 Patients

Patients harboring pathogenic variants in *CNGA3, CNGB3, GNAT2, PDE6C, PDE6H*, and *ATF6* genes, which are responsible for achromatopsia (ACHM), were recruited for this study. All participants were of Han ethnicity, and the age at symptom onset was less than 6 years. In total, 13 patients from 12 families were collected, among which patient 11 and patient 12 were from the same family. The study ran from January 2018 to January 2024 and was conducted in accordance with the Declaration of Helsinki. It received approval from the Medical Ethics Committee of Zhongshan Ophthalmic Center, Sun Yat-sen University (ID:2020KYPJ175). Informed consent was obtained from the parents or legal guardians of the participating children. The medical records were reviewed retrospectively. Due to the challenges posed by the non-cooperation of toddlers, conducting visual acuity and color testing was difficult. Therefore, diagnosis relied on clinical presentations, including early-onset infantile nystagmus, normal fundus appearance, optical coherence tomography (OCT) findings, and abnormal cone responses with normal or subnormal rod responses observed on flash electroretinogram (fERG) testing.

### 2.2 Ophthalmic examinations

Ophthalmic examinations, including the measurement of the best-corrected visual acuity (BCVA), refractive error, a biomicroscopy with a slit-lamp microscope, and fundus examinations, were performed. The wide-field fundus photographs were taken from each eye, centered on the optic nerve and macula (Optos 200Tx, Optos PLC, Dunfermline, Scotland, UK; RetCam, Clarity Medical Systems, Pleasanton, CA). The optical coherence tomography (VG200D; SVision Imaging, Ltd., Henan, China) was performed for children with dilated pupils. The fERG (Ret-eval, LKC, Gaithersburg, MD, USA) was recorded according to the International Guidelines of the International Society of Clinical Electrophysiology of Vision (ISCEV). For uncooperative pediatric patients, the OCT and fERG are conducted under sedation with chloral hydrate. Color vision tests were performed in some cases using an Ishihara pseudoisochromatic plate test.

### 2.3 Grading of outer retinal damage on OCT

According to the modified classification system (21, 22), the severity of outer retinal damage shown on the OCT was graded in three grades. Grade 1 was characterized by an unrecognizable interdigitation zone (IZ). Grade 2 was characterized by an unrecognizable IZ and an indistinct ellipsoid zone (EZ). Grade 3 was defined by both an unrecognizable IZ and an interrupted EZ.

### 2.4 DNA sample collection and whole exome sequencing

DNA was extracted from the peripheral whole blood of each child and their family members using the methods described in our previous study (23). Whole exome sequencing (WES) was performed on probands. Sanger sequencing was used to verify the genetic variants via next-generation sequencing and segregation analysis of available family members. Pathogenicity predictions were performed using SIFT^1^, Polyphen-2^2^, and REVEL. Allele frequencies were retrieved from the Genome Aggregation Database (gnomAD)^3^. Based on the genetic results and the clinical features, the etiological factors were reviewed, and the final diagnosis was provided. The pathogenicity of variants was defined based on the criteria of the American College of Medical Genetics and Genomics (ACMG) (24).

### 2.5 Statistical analysis

Statistical analyses were performed using the SPSS software (IBM, Armonk, NY, version 26). Continuous variables are reported as means ± standard deviations (SDs) or medians with interquartile ranges, depending on the distribution of the data. Generalized Estimating Equations (GEEs) were performed to adjust the inter-eye consistency in evaluating the correlation between the degree of outer retinal layer damage and genetic variants.

## 3 Results

### 3.1 Demographics and clinical features of ACHM in preschool children

Thirteen patients diagnosed with ACHM were included in the study, all involving both eyes. Table 1 provides an overview of their demographic and clinical characteristics. Among the participants, five were boys and eight were girls. The median age at the initial visit was 3 years (IQR 2.0–6.0), with a mean age at symptom onset of 0.88 ± 1.07 years. During their initial consultation, parents reported primary complaints, including nystagmus (46.15%, 6/13), photophobia (46.15%, 6/13), and poor visual acuity (30.8%, 4/13), with one patient presenting with reduced visual acuity (7.69.%, 1/13). The median spherical equivalent (SE) refraction was +2.13 diopter (D) (range: −5.50 ~ +4.38D). Six children can complete vision testing; the mean BCVAs, converted to LogMAR, were 1.02 ± 0.11. Color vision testing revealed complete color blindness.

**Table 1:** Main clinical findings in each achromatopsia patient.

ID	Gender	Age at symptom onset (years)	Age at initial presentation (years)	Reported major symptom	SE (D)		BCVA		Fundus		Outer retinal damage on OCT		Color vision test	
					OD	OS	OD	OS	OD	OS	OD	OS	OD	OS
1	F	0.3	6	Nystagmus	1.125	1.375	0.80	1.00	Normal	Normal	3	3	Complete	Complete
2	F	0.2	1	Nystagmus	2.125	1.375	NA	NA	Normal	Normal	3	2	NA	NA
3	M	2.0	6	Photophobia	3.125	2.625	1.00	1.00	Normal	Normal	NA	NA	Complete	Complete
4	M	0.2	3	Nystagmus	NA	NA	NA	NA	Normal	Normal	NA	NA	NA	NA
5	M	1.0	2	Photophobia	NA	NA	NA	NA	Normal	Normal	NA	NA	NA	NA
6	F	0.2	3	Nystagmus	2.125	2.25	1.00	1.22	Normal	Normal	1	2	NA	NA
7	F	0.2	3	Nystagmus	NA	NA	NA	NA	Normal	Normal	2	2	NA	NA
8	F	1.0	7	Photophobia	2.125	1.625	1.00	1.00	Normal	Normal	2	2	Complete	Complete
9	M	4.0	16	Poor vision	−2.875	−5.5	1.22	1.00	Pale optic disc, C/D 0.6	Pale optic disc, C/D 0.5	2	3	Complete	Complete
10	F	1.0	2	Nystagmus	4.375	4.25	NA	NA	Normal	Normal	3	3	NA	NA
11	F	0.5	6	Photophobia	NA	NA	1.00	1.00	Normal	Normal	2	2	Complete	Complete
12	M	0.5	3	Photophobia	NA	NA	NA	NA	Normal	Normal	3	3	NA	NA
13	F	0.3	2	Photophobia	NA	NA	NA	NA	Normal	Normal	NA	NA	NA	NA

SE, spherical equivalent; D, diopter; BCVA, best-corrected visual acuity; F, Female; M, Male; NA, not applicable; C/D, cup-to-disc ratio. Outer retinal damage grade: Grade 1 was characterized by an unrecognizable interdigitation zone (IZ). Grade 2 was characterized by an unrecognizable IZ and an indistinct ellipsoid zone (EZ). Grade 3 was defined by both an unrecognizable IZ and an interrupted EZ.

### 3.2 Retinal phenotypes

**Figure 1:**
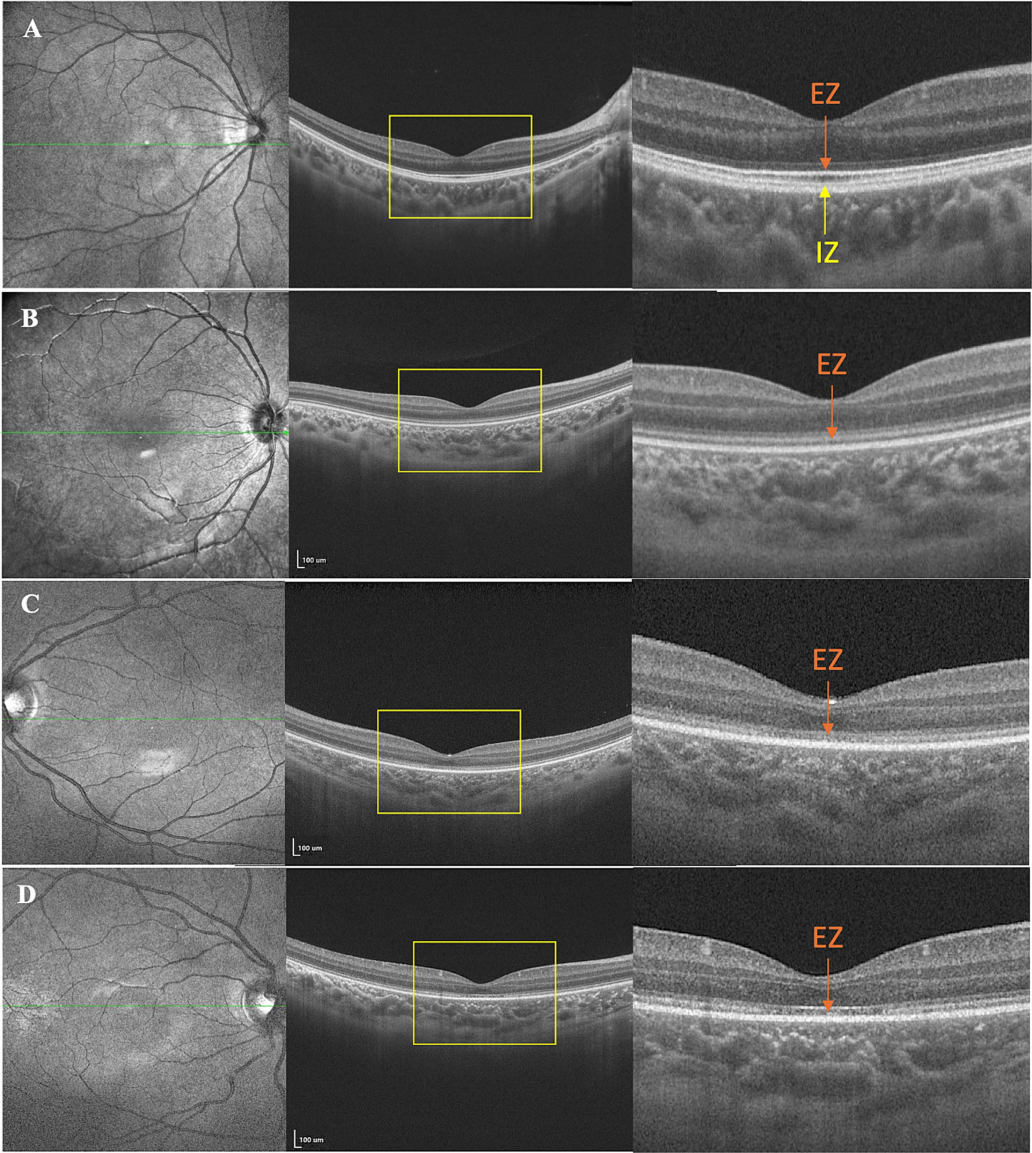
Representative images of multimodal ophthalmic imaging of patients showing outer retinal damage. **(A)** The healthy control. **(B)** Grade 1 was characterized by an unrecognizable interdigitation zone (IZ). **(C)** Grade 2 was characterized by both an unrecognizable IZ and an indistinct ellipsoid zone (EZ). **(D)** Grade 3 was defined by an unrecognizable IZ and an interrupted EZ.

All patients exhibited unremarkable anterior segment findings. Fundus photography detected no significant abnormalities except in patient 9, who presented bilaterally pale optic discs with a cup-to-disc ratio of 0.5. However, OCT imaging demonstrated variable foveal structural anomalies across the cohort. Four young children were excluded from OCT analysis due to non-compliance, leaving nine cooperative patients (18 eyes) for retinal layer evaluation. OCT revealed diffuse outer retinal disruptions localized to the foveal region, specifically affecting the IZ and EZ. Quantitative analysis of the 18 evaluated eyes demonstrated universal outer retinal layer involvement, stratified as grade 1 (5.6%, *n* = 1), grade 2 (50.0%, *n* = 9), and grade 3 (44.4%, *n* = 8) abnormalities (Table 1), with representative morphological features shown in Figure 1.

### 3.3 Genotypes

**Figure 2:**
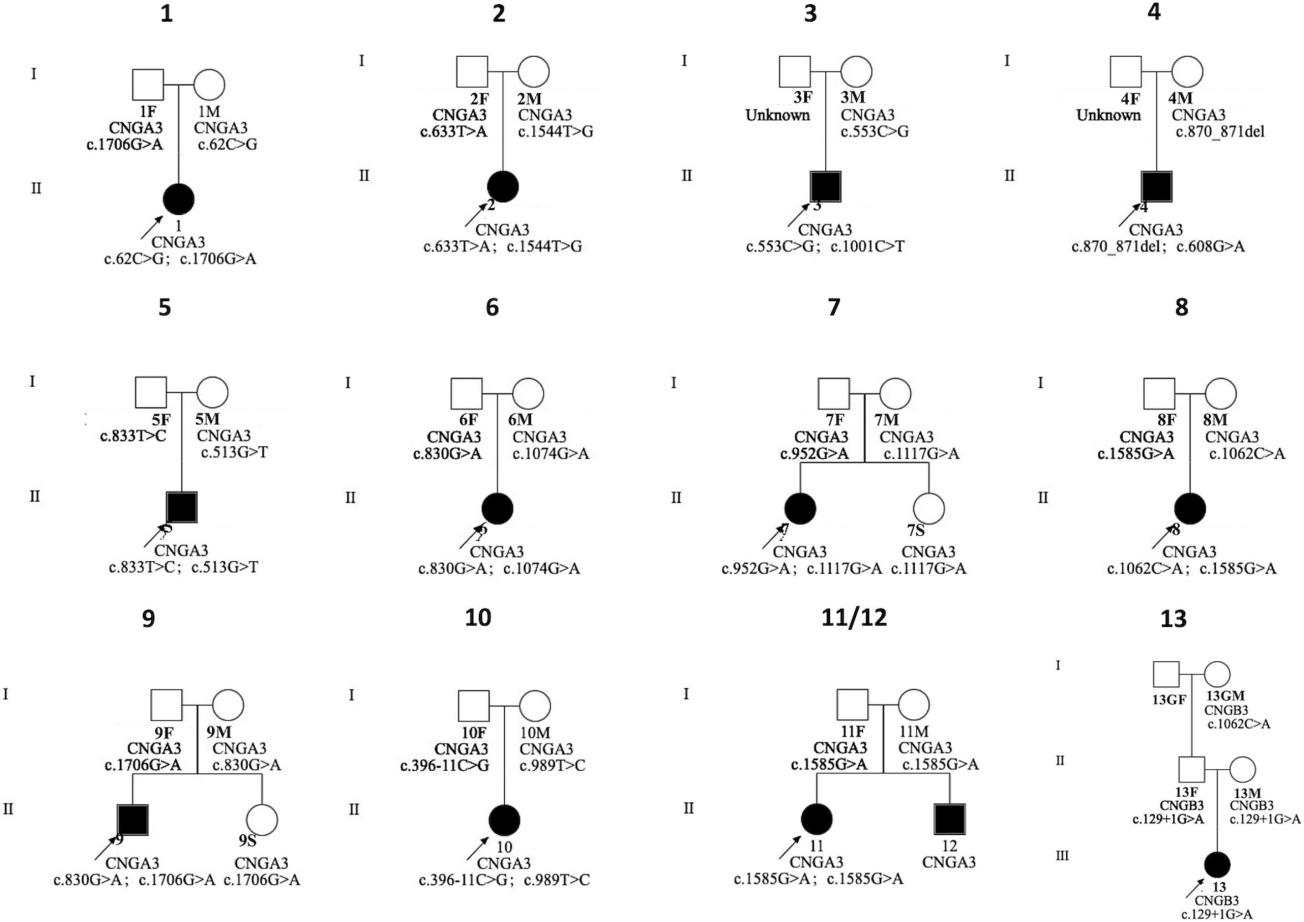
The pedigree of the families with genetic variants in *CNGA3* and *CNGB3* genes. F, father; GF, grandfather; GM, grandmother; M, mother; S, sister.

**Figure 3:**
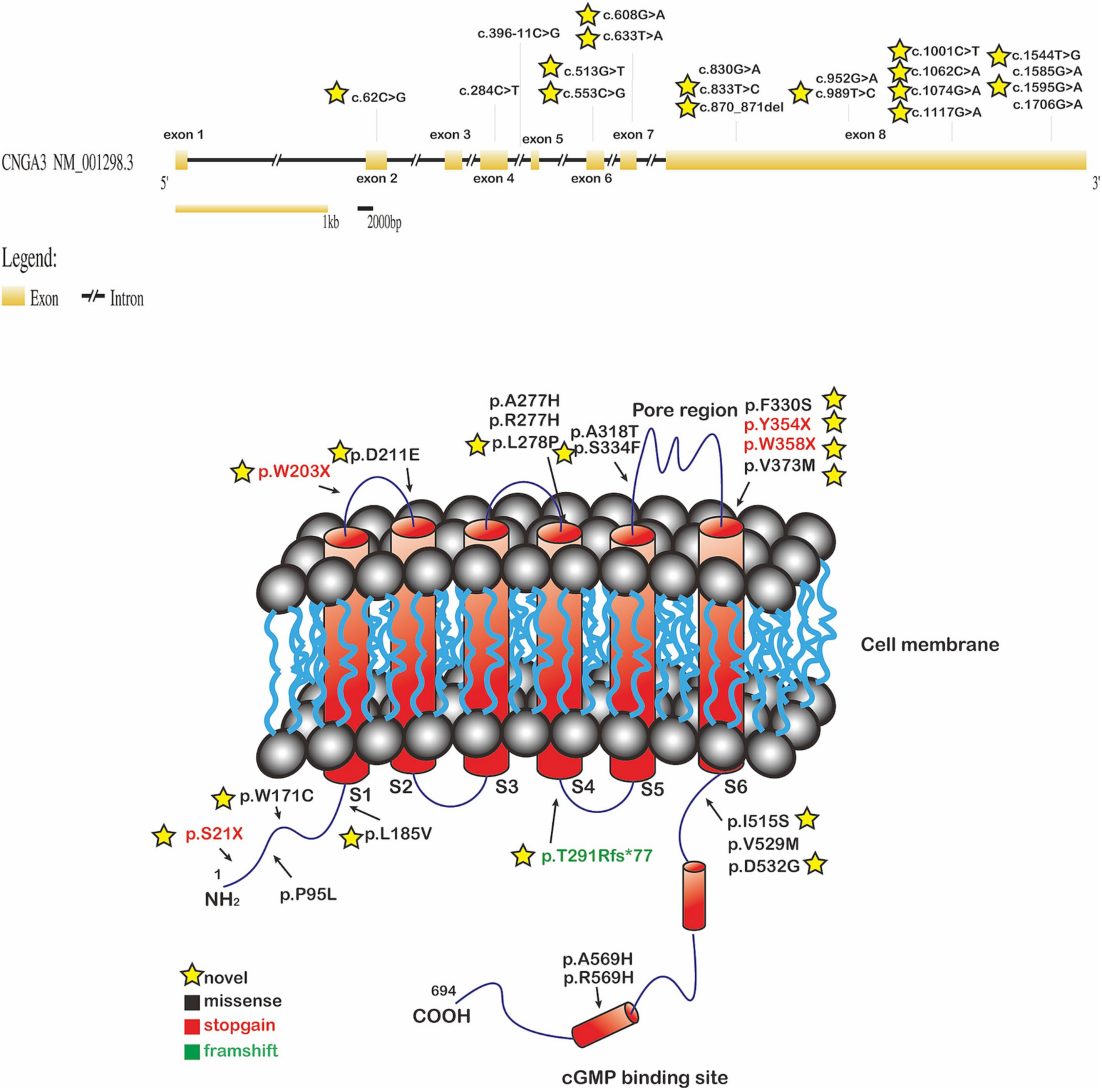
The *CNGA3* gene locus with its eight exons (GRCh38/hg38, NM_001298.2) was depicted at the top of the figure. Exons were indicated by the yellow rectangles. Introns were indicated by the black line. The locations of variants are shown in one of the diagrams of the *CNGA3* gene structure. To localize the genetic variants with respect to the proposed topological model of the *CNGA3*. The location of missense (black), stop gain (red), and frameshift (green) variants were shown on the protein. The yellow star indicated the novel variants; cGMP: cyclic guanosine monophosphate.

We identified 20 variants among the 13 patients. Notably, we found pathogenic variants in two common causative genes for ACHM (Table 2; Figures 2, 3). The causes of ACHM were defects in *CNGA3* in 12 patients (92.3%) and *CNGB3* in one patient (7.7%). Of these 13 patients, 12 were compound heterozygotes, and just one was a homozygote. Among the 20 identified variants, 55% (11/20) represented novel findings, with six classified as pathogenic/likely pathogenic per ACMG guidelines and five remaining variants of uncertain significance (VUS). The *CNGA3* variant spectrum comprised predominantly missense variants (68.4%, 13/19), followed by stop gain (21.1%, 4/19), frameshift (5.3%, 1/19), and splicing variants (5.3%, 1/19). We also found that three *CNGA3* variants showed up in multiple patients. The c.830G > A variant was in patients #6 and #9, c.1706G > A was in patients #1 and #9, and c.1585G > A was in patients #8, #11, and #12 (who is patient #11’s younger brother). Additionally, the sole patient with *CNGB3*-ACHM harbored a homozygous canonical splicing variant (c.129 + 1G > A).

**Table 2:** Genetic findings in the achromatopsia patients.

ID	Gene	Exon	Variants	Location	Allele Type	ACMG Criteria	ACMG Result	SIFT	Polyphen-2	REVEL	gnomAD	Inheritance	Reference
1	*CNGA3*	exon2	NM_001298: c.62C > G (p. Ser21*)	chr2:98986500	stopgain	PVS1, PM2, PM3	P	–	–	–	0.00002033	Mother	(31–33)
1	*CNGA3*	exon8	NM_001298: c.1706G > A (p. Arg569His)	chr2:99013339	missense	PM1, PM3, PP3, PP1	LP	PD	PD	PD	0.00002166	Father	(34)
2	*CNGA3*	exon7	NM_001298: c.633T > A (p. Asp211Gly)	chr2:99008393	missense	PM1, PM2	VUS	D	D	D	0	Father	Novel
2	*CNGA3*	exon8	NM_001298: c.1544T > G (p. lle515Ser)	chr2:99013177	missense	PM1, PM2	VUS	D	D	D	0	Mother	Novel
3	*CNGA3*	exon6	NM_001298: c.553C > G (p. Leu185Val)	chr2:99006224	missense	PM1, PM2, BP4	VUS	–	–	–	0.000004	Mother	Novel
3	*CNGA3*	exon8	NM_001298: c.1001C > T (p. Ser334Phe)	chr2:99012634	missense	PM1, PM2, PP3	VUS	PD	PD	PD	0	Unknown	(18, 32)
4	*CNGA3*	exon7	NM_001298: c.608G > A (p. Trp203*)	chr2:99008368	stopgain	PVS1, PM2, PP3	P	–	–	–	0	Unknown	Novel
4	*CNGA3*	exon8	NM_001298: c.870_871del (p. Thr291Argfs*77)	chr2:99012502	frameshift	PVS1, PM2, PP4	P	–	–	–	0	Mother	Novel
5	*CNGA3*	exon6	NM_001298: c.513G > T (p. Trp171Cys)	chr2:99006184	missense	PM1, PM2, PP3	VUS	D	D	D	0.000004	Mother	(31, 32)
5	*CNGA3*	exon8	NM_001298: c.833T > C (p. Leu278Pro)	chr2:99012466	missense	PM1, PM2, PP3	VUS	D	D	D	0.000004	Father	Novel
6	*CNGA3*	exon8	NM_001298.2: c.830G > A (p. Arg277His)	chr2:99012463	missense	PS1, PM2, PM3, PP3, PP5	P	D	D	D	0.000024	Father	(14, 35)
6	*CNGA3*	exon8	NM_001298.2: c.1074G > A (p. Trp358*)	chr2:99012707	stopgain	PVS1, PM2, PM3	P	–	–	–	0.000012	Mother	Novel
7	*CNGA3*	exon8	NM_001298.2: c.952G > A(p. Trp358*)	chr2:99012585	missense	PM1,PM2, PP3,PP4	LP	D	D	D	0.000012	Father	(14, 35)
7	*CNGA3*	exon8	NM_001298.2: c.1117G > A (p. Val373Met)	chr2:99012750	missense	PM2,PM3,PM5, PP3	LP	D	D	D	0.00004	Mother	Novel
8	*CNGA3*	exon8	NM_001298.2: c.1062C > A (p. Tyr354*)	chr2:99012695	stopgain	PVS1, PM2, PM3	P	–	–	–	0	Mother	Novel
8	*CNGA3*	exon8	NM_001298.2: c.1585G > A (p. Val529Met)	chr2:99013218	missense	PM1, PM2, PP3, PP5	LP	D	D	D	0.000067	Father	(32, 36–38)
9	*CNGA3*	exon8	NM_001298.3: c.830G > A (p. Arg277His)	chr2:99012463	missense	PS1, PM2, PM3, PP3, PP5	P	D	D	D	0.000024	Mother	(14, 35)
9	*CNGA3*	exon8	NM_001298.3: c.1706G > A (p. Arg569His)	chr2:99013339	missense	PS1, PP3, PP5	LP	PD	PD	PD	0.00002166	Father	(34)
10	*CNGA3*	intron4	NM_001298.3: c.396-11C > G(p.?)	–	splicing	PVS1, PM2, PM3, PP3	P	–	–	–	0	Father	(32)
10	*CNGA3*	exon8	NM_001298.3: c.989T > C (p. Phe330Ser)	–	missense	PS1, PM2, PM3, PP3, PP5	P	–	–	–	0	Mother	Novel
11	*CNGA3*	exon8	NM_001298.3: c.1585G > A (p. Val529Met)	chr2:99013218	missense	PS4, PM3, PP1, PP3	LP	D	D	D	0.0006	Father	(32, 36–38)
11	*CNGA3*	exon8	NM_001298.3: c.1595G > A (p. Asp532Gly)	chr2:99013228	missense	PM2, PM3, PP3	VUS	D	D	D	0	Mother	Novel
12	*CNGA3*	exon8	NM_001298.3: c.1585G > A (p. Val529Met)	chr2:99013218	missense	PS4, PM3, PP1, PP3	LP	D	D	D	0.0006	Father	(32, 36–38)
12	*CNGA3*	exon8	NM_001298.3: c.1595G > A (p. Asp532Gly)	chr2:99013228	missense	PM2, PM3, PP3	VUS	D	D	D	0	Mother	Novel
13	*CNGB3*	exon1	NM_019098.4: c.129 + 1G > A (p.?)	chr8:87755726	splicing	PVS1, PS1, PM3	P	–	–	–	0.000008	Father And Mother	(39)

D, damaging; PD, probably damaging; P, pathogenic; LP, likely pathogenic; VUS: Variants of Uncertain Significance. ACMG, American College of Medical Genetics and Genomics.

### 3.4 Genotype-phenotype correlation analysis

Eighteen eyes with OCT were included in further analysis of the association between genetic variants and outer retinal layer damage. Patients with genetic variants of *CNGA3* were divided into two groups based on whether their genetic variants could potentially lead to structural changes in the cyclic guanosine monophosphate (cGMP) binding site of the synthesized protein. In the cGMP group, none of the eyes were classified as grade 1 (0 eyes, 0.0%), while six eyes (42.9%) were classified as grade 2 and eight eyes (57.1%) were classified as grade 3, resulting in a total of 14 eyes. In contrast, among the other genetic variant groups, one eye (25.0%) was categorized as grade 1, three eyes (75.0%) were categorized as grade 2, and none of them fell into grade 3 (0.0%), with a total of four eyes. There were no statistically significant differences in the median age, BCVA, and SE between the two groups. The higher grade of outer retinal layer damage was shown in patients with genetic variants, potentially leading to structural changes in the cGMP binding site of the synthesized protein (*p* = 0.046, Table 3).

**Table 3:** Comparison of achromatopsia patients based on the presence or absence of cGMP binding site genetic variants.

	cGMP binding site genetic variants	Other genetic variants	*p*-value
Age (y)	6.00 (2.00, 7.00)	3.00 (3.00, 3.00)	0.44
SE (D)	1.50 (0.13, 2.66)	2.19 (2.13, 2.21)	0.40
BCVA	1.00 (1.00, 1.00)	1.11 (1.00, 1.14)	0.36
Outer retinal damage on OCT (eyes)	14	4	0.046
Grade 1	0	1	–
Grade 2	6	3	–
Grade 3	8	0	–

The results of the first three rows are presented as median (Lower Quartile, Upper Quartile) deviation. y, years; SE, spherical equivalent; D, diopter; BCVA, best-corrected visual acuity.

## 4 Discussion

In the present study, we analyzed the genotypes and phenotypes of 13 Chinese children with ACHM. This study described detailed clinical characteristics in much younger populations, especially the detailed outer retinal architecture. Compared to previous studies, this is crucial given the rapid and ongoing advancements in gene therapy for ACHM.

The mutational profiles of ACHM patients exhibit significant global heterogeneity. In the Netherlands, the prevalence of *CNGB3* gene variants in ACHM patients is substantial (87%) (13). *CNGA3* (38.1%) and PDE6C (38.1%) are the most prevalently mutated genes in Korean patients with ACHM. In the US and the UK, the main genetic variants in ACHM patients are *CNGA3* and *CNGB3*, with *CNGA3* being more prevalent, but the difference between the two is not significant. However, in our patient cohort, *CNGA3* variants were presented in 92.3% of patients, accounting for an extremely high proportion, of which missense and truncating variants accounted for 75% (18/24) and 25% (6/24), respectively. Zhang et al. have also documented that *CNGA3* was identified as the most common pathologic gene in Chinese ACHM patients (81.5%) (25). Phase I/II clinical trials for *CNGA3*-ACHM and *CNGB3*-ACHM are underway in the United States. The aforementioned results underscore the necessity for *CNGA3*-ACHM gene therapy.

We reported 11 novel gene variants in *CNGA3*, which are not documented in population databases, expanding the catalog of known potentially pathogenic variants. The genetic variants in the *CNGA3* gene were localized in the cytoplasmic NH2 terminus, the transmembrane helices, and the cGMP binding domain of the *CNGA3* protein, and all caused an amino acid change (26) (Table 3; Figure 3). Interestingly, the phenotypic consequences were severe in patients with variants, potentially leading to structural changes in the cGMP binding site of the synthesized protein. The possible mechanism might involve the overactivation of the CNG channel, which is reliant on the regulation of cGMP binding. Choi et al. reported similar findings, where patients with PDE6C variants exhibited more severe abnormalities in the outer retinal layer than those with variants in *CNGA3*, *CNGB3*, and GNAT2 (27). PDE6C has been identified as the gene encoding the alpha-prime subunits of cone cell phosphodiesterase, which is crucial in regulating cGMP synthesis (28).

Twelve of our patients (92.3%) showed normal manifestation in fundus photography, with notable abnormalities in OCT. With the aid of sedation, we were able to obtain OCT results in younger patients (the minimum examining age is 1.10 years). In Korean *CNGA3*-ACHM patients, 37.5% (3/8) displayed EZ disruption. Conversely, more severe photoreceptor degenerations were noted in Italian populations, where OCT identified the absence of EZ, the presence of a hypo-reflective zone, and outer retinal atrophy of 42.9% *CNGA3*-ACHM patients (3/7). This finding aligns with results from another study on American *CNGA3*-ACHM populations, where 44.4% (8/18) showed similar OCT findings, including the absence of EZ, hypo-reflective zone presence, and outer retinal atrophy (18). Compared to previous research, our cohort exhibited a milder degree of damage to the outer layers of the retina (21, 27). The reason for this observation might be attributed to the relatively younger age of our study population (median age 2.84 years old vs. 24.50 in X, 19.00 in X). With the continuous development of optical coherence tomography (OCT), we have detected more subtle OCT changes. Compared to previous studies (21, 22), our OCT grading system focuses on the IZ, which was not considered in past OCT grading systems. IZ, its location at the interface of the RPE and photoreceptor outer segments, implies a possible role in visual signal-related processes such as enhancing substance exchange for proper photoreceptor operation and visual signal transduction (29). We noted that when the ellipsoid zone is continuous, the IZ can be unrecognizable, which we designated as Grade 1. In Grade 2, besides the ellipsoid zone indistinct, patients also show IZ changes. Moreover, in Wu et al.’s case report, they also detected the interdigitation zone changes (30). Therefore, we propose a novel OCT grading system designed specifically for the evaluation of outer retinal damage in preschool children.

Our findings indicated that ACHM might progressively worsen with age, contrary to the previously held belief that it remains “stationary.” Crucially, in Chinese *CNGA3*-ACHM, OCT revealed structural damage of the outer retinal layers in early life, particularly in IZ and EZ, detectable as early as 1.0 years of age (median onset: 3.0 years; IQR 2.0–6.0). Early detection of subtle pathological changes in OCT scans and accurate grading can enable timely diagnosis and early identification of the disease, allowing children to start treatment immediately. Furthermore, in a mouse model with human CNGB3 packaged in an AAV8 capsid, functional rescue was demonstrated by ERG testing across different ages, yet older mice showed a poor response to the treatment (9). Collectively, these observations collectively underscore the critical need to advance the therapeutic window for gene therapy interventions.

This study has some limitations in its retrospective design. First, despite patients being encouraged to attend annual follow-up visits, the duration of follow-up varied among participants. Consequently, only cross-sectional data were obtainable for some patients or certain tests. Furthermore, due to the low prevalence of *CNGA3* and *CNGB3* patients, the sample size of 13 patients was relatively low. As a result, it was difficult to perform subgroup comparisons between the two genes. Finally, it is hard to conduct certain subjective tests, including color vision tests and visual acuity assessments, because of the young age of our cohort.

## 5 Conclusion

A better understanding of the disease course and progression is crucial for patient management. Our findings sustain the onset of ACHM in early life. OCTs are effective diagnostic tools for disease and can be conducted under sedation when necessary. A mild degree of damage to the outer layers of the retina and macular hypoplasia are typical changes in early-stage ACHM. Moreover, we reported 11 new genetic variants, contributing to the expansion of the disease variant spectrum; the severe degree of outer retinal damage was observed in patients with genetic variants, potentially leading to structural changes in the cGMP binding site of the synthesized protein. Ultimately, our research may aid in formulating guidelines for selecting patients and determining the optimal timing for interventions in upcoming gene replacement therapies.

## Data Availability

The datasets presented in this article are not readily available because of concerns regarding participant/patient anonymity. Requests to access the datasets should be directed to the corresponding author.

## Footnotes

^1^ http://sift.jcvi.org/www/SIFT_enst_submit.html

^2^ http://genetics.bwh.harvard.edu/pph2/

^3^ http://www.gnomad-sg.org

## References

[1] Ashtari M Cyckowski LL Monroe JF Marshall KA Chung DC Auricchio A. The human visual cortex responds to gene therapy-mediated recovery of retinal function. J Clin Invest. (2011) 121:2160–8. doi: 10.1172/JCI57377, PMID: 21606598 PMC3104779

[2] Ashtari M Zhang H Cook PA Cyckowski LL Shindler KS Marshall KA. Plasticity of the human visual system after retinal gene therapy in patients with Leber’s congenital Amaurosis. Sci Transl Med. (2015) 7:296ra110. doi: 10.1126/scitranslmed.aaa8791, PMID: 26180100 PMC4617524

[3] Fischer MD Michalakis S Wilhelm B Zobor D Muehlfriedel R Kohl S. Safety and vision outcomes of subretinal gene therapy targeting cone photoreceptors in Achromatopsia: a nonrandomized controlled trial. JAMA Ophthalmol. (2020) 138:643–51. doi: 10.1001/jamaophthalmol.2020.1032, PMID: 32352493 PMC7193523

[4] McKyton A Averbukh E Marks Ohana D Levin N Banin E. Cortical visual mapping following ocular gene augmentation therapy for Achromatopsia. J Neurosci. (2021) 41:7363–71. doi: 10.1523/JNEUROSCI.3222-20.2021, PMID: 34349002 PMC8412991

[5] Farahbakhsh M Anderson EJ Maimon-Mor RO Rider A Greenwood JA Hirji N. A demonstration of cone function plasticity after gene therapy in Achromatopsia. Brain. (2022) 145:3803–15. doi: 10.1093/brain/awac226, PMID: 35998912 PMC9679164

[6] Pang JJ Deng WT Dai X Lei B Everhart D Umino Y. Aav-mediated cone Rescue in a Naturally Occurring Mouse Model of Cnga3-Achromatopsia. PLoS One. (2012) 7:e35250. doi: 10.1371/journal.pone.0035250, PMID: 22509403 PMC3324465

[7] Banin E Gootwine E Obolensky A Ezra-Elia R Ejzenberg A Zelinger L. Gene augmentation therapy restores retinal function and visual behavior in a sheep model of Cnga3 Achromatopsia. Mol Ther. (2015) 23:1423–33. doi: 10.1038/mt.2015.114, PMID: 26087757 PMC4817879

[8] Komaromy AM Alexander JJ Rowlan JS Garcia MM Chiodo VA Kaya A. Gene therapy rescues cone function in congenital Achromatopsia. Hum Mol Genet. (2010) 19:2581–93. doi: 10.1093/hmg/ddq136, PMID: 20378608 PMC2883338

[9] Carvalho LS Xu J Pearson RA Smith AJ Bainbridge JW Morris LM. Long-term and age-dependent restoration of visual function in a mouse model of Cngb3-associated Achromatopsia following gene therapy. Hum Mol Genet. (2011) 20:3161–75. doi: 10.1093/hmg/ddr218, PMID: 21576125 PMC3140821

[10] Reichel FF Michalakis S Wilhelm B Zobor D Muehlfriedel R Kohl S. Three-year results of phase I retinal gene therapy trial for Cnga3-mutated Achromatopsia: results of a non randomised controlled trial. Br J Ophthalmol. (2022) 106:1567–72. doi: 10.1136/bjophthalmol-2021-319067, PMID: 34006508

[11] Alexander JJ Umino Y Everhart D Chang B Min SH Li Q. Restoration of cone vision in a mouse model of Achromatopsia. Nat Med. (2007) 13:685–7. doi: 10.1038/nm1596, PMID: 17515894 PMC3985124

[12] Michalakis S Muhlfriedel R Tanimoto N Krishnamoorthy V Koch S Fischer MD. Restoration of cone vision in the Cnga3−/− mouse model of congenital complete lack of cone photoreceptor function. Mol Ther. (2010) 18:2057–63. doi: 10.1038/mt.2010.149, PMID: 20628362 PMC2997579

[13] Thiadens AA Slingerland NW Roosing S van Schooneveld MJ van Lith-Verhoeven JJ van Moll-Ramirez N. Genetic etiology and clinical consequences of complete and incomplete Achromatopsia. Ophthalmology. (2009) 116:1984–1989.e1. doi: 10.1016/j.ophtha.2009.03.053, PMID: 19592100

[14] Fahim AT Khan NW Zahid S Schachar IH Branham K Kohl S. Diagnostic fundus autofluorescence patterns in Achromatopsia. Am J Ophthalmol. (2013) 156:1211–1219.e2. doi: 10.1016/j.ajo.2013.06.033, PMID: 23972307

[15] Greenberg JP Sherman J Zweifel SA Chen RW Duncker T Kohl S. Spectral-domain optical coherence tomography staging and autofluorescence imaging in Achromatopsia. JAMA Ophthalmol. (2014) 132:437–45. doi: 10.1001/jamaophthalmol.2013.7987, PMID: 24504161 PMC4423754

[16] Baxter MF Borchert GA. Gene therapy for Achromatopsia. Int J Mol Sci. (2024) 25:9739. doi: 10.3390/ijms25179739, PMID: 39273686 PMC11396370

[17] Michalakis S Gerhardt M Rudolph G Priglinger S Priglinger C. Achromatopsia: genetics and gene therapy. Mol Diagn Ther. (2022) 26:51–9. doi: 10.1007/s40291-021-00565-z, PMID: 34860352 PMC8766373

[18] Sundaram V Wilde C Aboshiha J Cowing J Han C Langlo CS. Retinal structure and function in Achromatopsia: implications for gene therapy. Ophthalmology. (2014) 121:234–45. doi: 10.1016/j.ophtha.2013.08.017, PMID: 24148654 PMC3895408

[19] Hirji N Aboshiha J Georgiou M Bainbridge J Michaelides M. Achromatopsia: clinical features, molecular genetics, animal models and therapeutic options. Ophthalmic Genet. (2018) 39:149–57. doi: 10.1080/13816810.2017.1418389, PMID: 29303385

[20] Kohl S Hamel C. Clinical utility gene card for: Achromatopsia - update 2013. Eur J Hum Genet. (2013) 21:1–3. doi: 10.1038/ejhg.2013.44, PMID: 23486539 PMC3798849

[21] Brunetti-Pierri R Karali M Melillo P Di Iorio V De Benedictis A Iaccarino G. Clinical and molecular characterization of Achromatopsia patients: a longitudinal study. Int J Mol Sci. (2021) 22:1681. doi: 10.3390/ijms22041681, PMID: 33562422 PMC7914547

[22] Aboshiha J Dubis AM Cowing J Fahy RT Sundaram V Bainbridge JW. A prospective longitudinal study of retinal structure and function in Achromatopsia. Invest Ophthalmol Vis Sci. (2014) 55:5733–43. doi: 10.1167/iovs.14-14937, PMID: 25103266 PMC4161486

[23] Tang M Ding X Li J Hu A Yuan M Yang Y. Novel mutations in Fzd4 and phenotype-genotype correlation in Chinese patients with familial exudative vitreoretinopathy. Mol Vis. (2016) 22:917–32. PMID: 27555740 PMC4968609

[24] Richards S Aziz N Bale S Bick D Das S Gastier-Foster J. Standards and guidelines for the interpretation of sequence variants: a joint consensus recommendation of the American College of Medical Genetics and Genomics and the Association for Molecular Pathology. Genet Med. (2015) 17:405–24. doi: 10.1038/gim.2015.30, PMID: 25741868 PMC4544753

[25] Sun W Zhang Q. Diseases associated with mutations in Cnga3: genotype-phenotype correlation and diagnostic guideline. Prog Mol Biol Transl Sci. (2019) 161:1–27. doi: 10.1016/bs.pmbts.2018.10.002, PMID: 30711023

[26] Kaupp UB Seifert R. Cyclic nucleotide-gated ion channels. Physiol Rev. (2002) 82:769–824. doi: 10.1152/physrev.00008.2002 12087135

[27] Choi YJ Joo K Lim HT Kim SS Han J Woo SJ. Clinical and genetic features of Korean patients with Achromatopsia. Genes (Basel). (2023) 14:519. doi: 10.3390/genes14020519, PMID: 36833446 PMC9957537

[28] Georgiou M Robson AG Singh N Pontikos N Kane T Hirji N. Deep phenotyping of Pde6c-associated Achromatopsia. Invest Ophthalmol Vis Sci. (2019) 60:5112–23. doi: 10.1167/iovs.19-27761, PMID: 31826238 PMC6905659

[29] Staurenghi G Sadda S Chakravarthy U Spaide RF. International nomenclature for optical coherence tomography P. Proposed lexicon for anatomic landmarks in Normal posterior segment spectral-domain optical coherence tomography: the in*Oct consensus. Ophthalmology. (2014) 121:1572–8. doi: 10.1016/j.ophtha.2014.02.023, PMID: 24755005

[30] Wu S Yu Y Wang Y Zhang L Fang X Ye P. Novel Atf6 homozygous variant in a Chinese patient with Achromatopsia. Ophthalmic Genet. (2024) 45:153–8. doi: 10.1080/13816810.2024.2322643, PMID: 38419580

[31] Liang X Dong F Li H Li H Yang L Sui R. Novel Cnga3 mutations in Chinese patients with Achromatopsia. Br J Ophthalmol. (2015) 99:571–6. doi: 10.1136/bjophthalmol-2014-305432, PMID: 25637600

[32] Li S Huang L Xiao X Jia X Guo X Zhang Q. Identification of Cnga3 mutations in 46 families: common cause of Achromatopsia and cone-rod dystrophies in Chinese patients. JAMA Ophthalmol. (2014) 132:1076–83. doi: 10.1001/jamaophthalmol.2014.1032 24903488

[33] Johnson S Michaelides M Aligianis IA Ainsworth JR Mollon JD Maher ER. Achromatopsia caused by novel mutations in both Cnga3 and Cngb3. J Med Genet. (2004) 41:e20. doi: 10.1136/jmg.2003.011437, PMID: 14757870 PMC1735666

[34] Li FF Huang XF Chen J Yu XD Zheng MQ Lu F. Identification of novel mutations by targeted exome sequencing and the genotype-phenotype assessment of patients with Achromatopsia. J Transl Med. (2015) 13:334. doi: 10.1186/s12967-015-0694-7, PMID: 26493561 PMC4618873

[35] Wissinger B Gamer D Jagle H Giorda R Marx T Mayer S. Cnga3 mutations in hereditary cone photoreceptor disorders. Am J Hum Genet. (2001) 69:722–37. doi: 10.1086/323613, PMID: 11536077 PMC1226059

[36] Zelinger L Greenberg A Kohl S Banin E Sharon D. An ancient autosomal haplotype bearing a rare Achromatopsia-causing founder mutation is shared among Arab Muslims and oriental Jews. Hum Genet. (2010) 128:261–7. doi: 10.1007/s00439-010-0846-z, PMID: 20549516

[37] Nishiguchi KM Sandberg MA Gorji N Berson EL Dryja TP. Cone Cgmp-Gated Channel mutations and clinical findings in patients with Achromatopsia, macular degeneration, and other hereditary cone diseases. Hum Mutat. (2005) 25:248–58. doi: 10.1002/humu.20142, PMID: 15712225

[38] Kohl S Marx T Giddings I Jagle H Jacobson SG Apfelstedt-Sylla E. Total Colourblindness is caused by mutations in the gene encoding the alpha-subunit of the cone photoreceptor Cgmp-gated Cation Channel. Nat Genet. (1998) 19:257–9. doi: 10.1038/935, PMID: 9662398

[39] Huang L Zhang Q Li S Guan L Xiao X Zhang J. Exome sequencing of 47 Chinese families with cone-rod dystrophy: mutations in 25 known causative genes. PLoS One. (2013) 8:e65546. doi: 10.1371/journal.pone.0065546, PMID: 23776498 PMC3679152

